# Exploring the Acceptability of HIV Testing in the UK Dental Setting: A Qualitative Study

**DOI:** 10.3390/dj12080246

**Published:** 2024-08-02

**Authors:** Janine Yazdi-Doughty, Anthony J. Santella, Stephen Porter, Richard G. Watt, Fiona Burns

**Affiliations:** 1 Department of Epidemiology & Public Health, Faculty of Pop Health Sciences, University College London, London WC1E 7HB, UK; 2 School of Dentistry, University of Liverpool, Pembroke Place, Liverpool L3 5PS, UK; 3 Marion Peckham Egan School of Nursing & Health Studies, Department of Public Health, Fairfield University, Fairfield, CT 06824, USA; asantella@fairfield.edu; 4 Eastman Dental Institute, Faculty of Medical Sciences, University College London, London NW3 2PF, UK; 5 Institute for Global Health, University College London, London NW3 2PF, UK

**Keywords:** dentistry, prevention, public health

## Abstract

HIV point of care testing (POCT) is a common approach to expanding testing into non-specialised settings. Dental services have untapped potential to screen for health conditions including HIV. However, the perspectives of UK dental patients, dental professionals, and people with HIV are unknown. Ten focus groups were undertaken with dental patients, professionals, and people with HIV. The Framework method was used to analyse the qualitative data. Six themes were generated from the focus group data. The themes explored perceptions of HIV, the purpose, appropriateness, and acceptability of HIV testing in dental settings, and new processes that would need to be established in order to successfully implement point of care HIV testing in UK dental settings. Training needs were identified including communication skills and updates to current knowledge about HIV. HIV testing in dental settings is generally acceptable to dental patients, dental professionals, and PWH. However, of concern were logistical challenges and the risk of patients surprised at being offered an HIV test during a visit to the dentist. Nonetheless, the public health benefits of the intervention were well understood, i.e., early detection of HIV and initiation of treatment to improve health outcomes. Dental teams were able to generate novel solutions that could help to overcome contextual and logistical challenges to implementing HIV testing in dental settings.

## 1. Background

Despite a downward trend in the numbers of people with HIV (PWH), late diagnosis of HIV remains a persistent public health concern. To increase the identification of people with undiagnosed HIV, HIV testing has been expanded into non-traditional healthcare and community settings. Expanded HIV testing normalises testing and enables testing to reach people not accessing specialised sexual health services. HIV point of care testing (POCT) is a common approach to expanding testing into non-specialised settings. However, outside of antenatal care, uptake of HIV testing guidance in non-traditional settings has generally been poor. Nonetheless, these settings can be both efficacious and acceptable alternative means of testing [1,2]. Historically, dentistry was at the forefront of HIV/Acquired Immune Deficiency Syndrome (AIDS) detection. Several conditions associated with AIDSpresent with oral manifestations including oral candidiasis, pseudomembranous candidiasis, oral hairy leukoplakia, Kaposi sarcoma, and erythematous candidiasis [3]. Though these conditions are infrequently seen in the era of antiretroviral therapy (ART), dentists have a professional obligation to identify and discuss any oral signs indicative of AIDS [4]. 

At the time of writing, no UK policies explicitly recommend dental settings as a site for expanded HIV testing. Most HIV testing in dental settings has taken place in dental hospitals, schools, and community clinics in the USA and Canada [5,6,7,8,9]. However, in the UK, expanded testing is recommended in hospital and general medical practice settings in “high” and “extremely high” HIV prevalence areas; in these areas, expanded HIV testing can be a cost-effective approach [10,11]. According to the National Institute for Health and Care Excellence (NICE), an area has a high HIV prevalence if the diagnosed HIV rate is between 2 and 5 per 1000 people aged 15 to 59. If the rate is 5 or more per 1000 people, the prevalence is considered extremely high.

Dental services have untapped potential to screen for chronic diseases. By performing POCT alongside dental care, patients can avoid additional appointments and waiting times at other services, thereby enhancing convenience [12]. Dental professionals hold an ideal position to assume an expanded role in screening [13,14]. For example, they have the training to check for serious health conditions, including oral cancer, and understand referral processes to secondary care [15]. UK studies have explored the potential role of dental teams in screening for conditions such as diabetes, cardiovascular disease, and human papillomavirus and associated risk factors [16,17]. These studies have demonstrated the feasibility of offering and performing health screening in dental settings and the referral of cases for onward care Dental settings may also present an opportunity to access patient groups who are not presenting elsewhere for HIV testing [18]. 

However, dental professionals have been reported to treat people with HIV differently when compared to people with other chronic and infective diseases [19]. HIV has long been associated with stigma and discrimination within dental practice and reluctance to treat people with HIV [20,21]. For example, dental professionals in US private practice have been reluctant to test for HIV because HIV- and HIV testing-related stigma is perceived as being bad for business [22]. Nonetheless, patients’ perspectives of HIV testing in dental settings are overwhelmingly positive in the US and Canada [23]. Whether HIV testing is appropriate and acceptable in dental settings from the perspective of UK dental patients and dental professionals has not previously been explored. Further, no previous study have explored the views of PWH about testing for HIV in dental settings. 

Therefore, the aim of this study was to understand the perceptions of UK dental professionals, dental patients, and PWH towards HIV testing in dental settings including its appropriateness and acceptability. Secondly, the study aimed to understand what factors are important to facilitate the implementation of HIV testing in UK dental settings.

## 2. Methods

Ethical approval was granted by the Essex, East of England, National Health Service Research Ethics Committee (IRAS 221512). Each study recruitment site underwent local research and development (R&D) approvals prior to study commencement.

### 2.1. Patient and Public Involvement 

Patient and public involvement (PPI) was embedded throughout the study; details of the full scope of PPI have been published elsewhere [24]. The topic guide resources were developed with the support of PWH and dental patients; all were regular attenders to dental practice. The first version of the topic guide was developed by JD and the PhD supervisory team FB, RGW and SP. Subsequently, both dental patient and professional topic guides were reviewed by a stakeholder group. The group included three PWH, two people with lived experience of homelessness, and two dental professionals/dental researchers. Thereafter, the topic guides then underwent piloting. The patient topic guide was developed further by a pilot focus group with three dental patients. The topic guide was dissected, the individual questions were rearranged. Superfluous questions were eliminated and additional questions were added. 

Additions to the patient topic guide included the following:

1. Asking dental patients about their knowledge of links between oral health and HIV.
2. Asking whether HIV testing would have an impact on how patients viewed their dentist

The dental professional topic guide underwent a pilot with eight postgraduate dentists studying University College London. 

Additions to the dental professional topic guide included the following questions:

1. Asking dental professionals how they would prefer to be remunerated and Incentivized to test for HIV
2. Asking dental professionals when they would prefer to perform the test—before appointment, during appointment, at the end of appointment and understand why.

### 2.2. Study Method

This study employed a qualitative methodology, which involved undertaking focus groups with dental patients, professionals and people with HIV. The outcome of the study was themes relating to perceptions of HIV testing in dental settings, including acceptability and appropriateness and perspectives on what factors are important to facilitate the implementation of HIV testing in UK dental settings.

### 2.3. Sampling and Recruitment 

The study employed a maximum variation sampling strategy; this strategy was adopted to ensure a diverse range of perspectives. In advance of this study, a systematic review was undertaken that informed the selection of participant characteristics by identifying factors known to be likely to influence perspectives on the appropriateness of HIV testing in dental settings. Dental professionals at the respective practices supported the recruitment of dental patients by providing verbal information, displaying posters, and distributing flyers. Four types of dental settings in the UK were targeted for recruitment:

(1) National Health Service (NHS) general dental practice (GDP)
(2) Private GDP
(3) Community Dental Services (CDS)
(4) Dental hospital.

Dental settings were identified through existing professional networks of the research team and through colleagues in sexual health services. Dental practice principals in GDP, clinical directors in CDS and service leads in hospital were contacted by email (JD) to request participation of staff and patients. 

#### 2.3.1. Dental Professionals

Selection criteria for dental professionals included years post qualification, service provider role (e.g., dentist, dental therapist, dental nurse, and receptionist) and dental care setting in which they worked (e.g., private and NHSGDP, community and hospital dental services). A provisional sample size of 24–32 dental team members was considered sufficient to gain insight from all dental roles and levels of experience across the various dental settings and to provide sufficient richness to answer the research question.

Dental professionals were recruited from dental settings located in areas of high or extremely high HIV prevalence. 

Once confirmation of participation was received and R&D processes were completed, dental professionals were identified through key contacts within each service who coordinated staff attendance at focus groups conducted in their respective dental settings during lunch hour. 

#### 2.3.2. Dental Patients 

Selection criteria for dental patients included age group, gender, and dental setting attended. Ethnicity and markers of socioeconomic status were not an explicit sampling criterion. The dental from which participants were recruited (e.g., homeless dental service, NHS GDP, and private GDP) generated an inherent diversity of socioeconomic status. Dental practice recruitment siteswere all based in Greater London in areas of high or extremely high HIV prevalence. 

Inclusion criteria specified people aged 18 years and over who met the sampling criteria above. People with insufficient spoken English to consent to and participate in focus groups and people lacking the capacity to consent were excluded from the study. Dental patients were recruited from their respective dental practices with support from dental practice staff and a clinical research nurse. 

A sample size of 20–30 dental patients was considered sufficient to achieve maximum variation in age and gender across the dental sites. Patients who indicated a willingness to participate in the focus groups left their details with the reception at each dental setting or used the details on the information sheets provided to contact the study team. As an incentive for participation, each participant, including service providers, was offered a GBP 20 gift voucher.

#### 2.3.3. PWH

Selection criteria for PWH included age and gender. A sample of 8–10 PWH was considered sufficient to obtain maximum variation in views. Selection criteria was based on age group and gender of PWH. For the purposes of this PhD study, PWH were identified and recruited through the Public Health Englang Positive Voices study distribution list [25]. This is a national cross-sectional probability survey of PWH accessing HIV care in England and Wales [26]. The Positive Voices survey was distributed at HIV clinics between January and September 2017 and was completed by a representative sample of 4422 PWH. The Positive Voices distribution list included respondents who were willing to participate in future research and had provided their contact details for this purpose. The Public Health England HIV Surveillance team supported the recruitment process by emailing participants registered to the database. NHS Health Research Authority granted approval for this recruitment strategy. People on the distribution list were emailed with study information. Demographic data were requested from those who wished to participate. The responses were reviewed, and those whose demographic information matched the sampling criteria for PWH were invited to participate in the study. All other respondents were sent an email of thanks for taking the time to respond.

### 2.4. Focus Group Conduct

Prior to each focus group, participants were sent the study information sheet and signposting information. Informed consent was obtained at the start of each session. The sessions took place in locations located near to or familiar to the participants. Dental professional focus groups took place in dental practices. Patient focus groups took place in hired, private community spaces or in University College London facilities. PWH sessions were conducted in a private room in their usual sexual health service. Sessions were recorded using a password-protected device and transcribed verbatim using a secure upload process to a University College London-approved transcription service. Focus group sessions were structured using topic guides administered by a lead facilitator supported by a co-facilitator. 

Broadly, the topics covered included the following:

○ Perceptions of HIV infection and understanding of current concepts such as undetectable = untransmissible (U = U)
○ Perceptions of the appropriateness of HIV testing in the dental setting and the scope of practice of dental professionals.
○ Anticipating patient responses to the offer of an HIV test.
○ Identifying challenges and opportunities for HIV testing in dental settings.
○ Identifying ways in which HIV testing could be successfully integrated into dental settings.
○ Anticipating ways to maximise HIV test uptake in the dental setting.

Focus groups continued until inductive thematic saturation had been achieved, i.e., new analytical concepts had ceased to emerge from the data and their was sufficient richness of data to answer the research question [27].

### 2.5. Researcher Reflexivity 

Attempts to ensure reflexivity in this study included reflecting upon positionality, including professional roles and personal identities, of the lead facilitator (JD), i.e., PhD student, dentist, and a cisgender, heterosexual White woman. Transparency was maintained by having a co-facilitator present during each focus group session. We recognised that HIV is stigmatised in Black African culture and might stymie conversations in a predominantly White group of participants. A Black African research assistant attended the PWH focus group; his presence helped to encourage Black African participants with HIV to share their experiences. Other facilitators included a man with lived experience of homelessness who attended the focus group for homeless people, a sexual health consultant, and research assistants from the Clinical Research Network. 

### 2.6. Data Analysis 

The Framework method was used to analyse the focus group transcripts, which utilised the six-step process described by Richie and Lewis: Transcription, familiarisation, coding, developing a working analytical framework, applying the framework, and charting data into the framework matrix [28]. This method was selected because it was sufficiently complex to accommodate the representation of theory within the framework, provided plain English summaries of data that enabled collaborative working between medical and lay persons, and was suitable for use where there was broad agreement about the nature of the phenomena of interest. The framework approach enabled novel codes and themes to be generated through inductive processes. Copies of the original transcripts were made available to JD, RGW and FB for review and coding; the development of the coding framework was a collaborative process. 

## 3. Findings

Ten focus groups were undertaken with a total of 59 participants. Participants included 12 dental patients (group size ranged between 3 and 7), seven PWH, and 40 service providers (group size range between 4 and 13). Twice as many males than females took part in the dental patient groups, and 70% of participants were aged 25–59 (Table 1). Dental professional participants were 80% female, and most were dentists or dental nurses (Table 2). 

Six themes were generated from the focus group data. The themes explored perceptions of HIV, the purpose, appropriateness, and acceptability of HIV testing in dental settings and new processes that would need to be established in order to implement testing. These are described in detail in the following sections (Table 3 and Supplemental Table S1). 

### 3.1. Perceptions of HIV and Implications for HIV Poct Acceptability

Most participants were aware of the positive impact of early diagnosis and initiation of ART for HIV outcomes. Dental professionals were positive about testing for HIV and providing dental care for PWH. However, there were notable gaps in HIV knowledge, which contributed to uncertainty about dental professionals’ competency to deliver HIV POCT. No dental team members or patients explicitly mentioned U = U or were willing to assert that an undetectable HIV viral load prevented transmission of HIV. 

Dental professionals and patients did not perceive HIV as an urgent public health priority compared with other more prevalent health conditions, such as diabetes, primarily because of the decreasing incidence and increasing manageability of HIV. Older dental patients described the change in the media from headlines describing HIV as a death sentence to a condition that had disappeared “off the radar” [Male, dental patient, private dental practice].

HIV testing at the dentist had the potential to make people feel surprised and uncertain about the purpose of POCT or fearful of the implications of a reactive HIV test result. However, many participants described the decision as a pragmatic one. 

The perceived seriousness of HIV was predominantly related to stigma, outdated stereotypes, the social ramifications of a diagnosis, and historical ideas about outcomes for PWH, which still permeated the HIV discourse. PWH felt that within the general population, “there’s a perception of good AIDS and bad AIDS. Like your transfusions that’s good and sex and IV drug use are the bad AIDS”. [Male with HIV, M2]. 

Some dental patients and dental professionals explained that the inference of HIV stereotypes and risk-taking behaviours might make patients feel offended when being offered an HIV test. 

### 3.2. Understanding the Purpose of HIV POCT in the Dental Setting

All participants perceived dental settings as well-suited to motivate patients to accept HIV testing during opportunistic encounters. The reasons were two-fold:

(1) the convenience of testing during routine dental appointments, thereby avoiding a separate appointment at a sexual health clinic (and the embarrassment or stigma of attending a sexual health clinic),
(2) the regularity of dental attendance could facilitate earlier diagnosis and referral for treatment.

Dental professionals and dental patients described the intervention’s unique opportunity to screen a cohort of asymptomatic people who may unknowingly be living with HIV. PWH were great advocates for opportunistic screening and shared their own stories of unexpected opportunistic diagnoses. Some dental professionals and patients thought that non-reactive test results provided reassurance for patients and an opportunity for dental professionals to offer health promotional advice.

A motivating factor for dental patients to test for HIV was reframing testing as part of overall wellness. Many dental patient participants strongly believed that dental care contributed to overall general health and wellbeing. Some dental professionals explained that though some patients may appreciate holistic care, others may want their dentist to stick to caring for their teeth.

### 3.3. Appropriateness of Dental Professionals to Provide HIV POCT

Participants across all focus groups strongly felt that the most challenging and yet essential part of implementing HIV testing in the dental setting was the effective management of reactive test results. There were three key threads of concern:

1. The first was the ability of dental professionals to break bad news appropriately and to manage the subsequent emotional response from the patient.
2. The second was the process of maintaining confidentiality, addressing questions, signposting, rapid referral, and linkage to care for confirmatory testing.

Both of the above concerns centred on minimising the distress and confusion for patients in the aftermath of a reactive POCT result.

3. The third concern was strongly voiced by PWH who felt that it was important that following an HIV diagnosis, patients were not stigmatised or treated differently.

Dental professionals felt anxious that HIV testing could negatively impact the patient–practitioner relationship, thereby impacting on business. HIV testing was generally perceived by dental professionals as incongruent with current dental practice. However, relevant transferrable skills were identified, e.g., communication. PWH and dental patients generally felt comfortable with the ability of the dental team to provide HIV testing so long as they were skilled members of the dental team and not part of the administrative staff. However, there were concerns about confidentiality, empathy and the risk of dental professionals treating people with HIV differently after a diagnosis. 

Patients explained that continuity of care, familiarity, and trust may help to reduce awkwardness when discussing HIV testing. Some PWH described how the customer culture inherent in private dental practice had enabled them to achieve a high quality of care from their dentist, irrespective of their HIV status. PWH felt that a relaxed, non-judgmental, and knowledgeable approach was likely to support HIV testing.

### 3.4. Appropriateness of Dental Settings for POCT

The dental practice environment offered several supportive factors to HIV testing. 

All participants felt that testing in inner-city dental practices and multi-disciplinary dental and health clinics where patients were younger, experiencing homelessness or misusing substances would increase the relevance of HIV testing. One colleague who worked between community dental service and general dental practice felt that the former would be a more supportive environment for HIV testing and did not have treatment targets. There were concerns about HIV testing interfering with the provision of dental care in general dental practice. All dental professionals described competing priorities such as limited clinical time and patient waiting times. Remuneration or incentivisation was a lower priority compared to the other competing logistical issues, and dental professionals were reluctant to discuss the specifics of reimbursement. 

A key concern from dental professionals and patients in all settings was having insufficient time to manage a reactive test result, which could compromise patient management and lead to increased waiting times for other patients. Dental professionals mentioned limited physical space as a prohibitive factor. A lack of spare rooms, open clinic bays, and reception areas made discrete conversations challenging. Closed dental surgeries offered a degree of anonymity that might motivate patients to accept testing. 

Team working and inter-professional collaborations encouraged positive attitudes towards HIV testing. In CDS, senior dental nurses were willing to deliver testing, describing their relevant skillset as including sedation training and blood pressure monitoring. Dental nurses in NHS and private GDP expressed reluctance to provide testing because they worried about being perceived as insufficiently qualified and that patients would not respond well to their involvement. Most dentists were confident that dental nurses could deliver testing and valued their support. Most dental patients and PWH were receptive to the idea of appropriately trained dental nurses testing for HIV. However, private dental patients preferred the testing to be performed by their regular dentist. 

### 3.5. Acceptability of the POCT Intervention in Dental Settings 

Service providers were concerned that offering POCT would provoke anxiety or embarrassment or elicit angry or aggressive responses. Therefore, discretion was of the utmost importance. All participants described the importance of patients not feeling obligated or coerced to have an HIV test. Factors that might impact patients’ ability to make an informed decision about HIV testing include language barriers, mental health issues, and anxiety. 

To counter patient anxiety about POCT, dental professionals suggested using positive language, a casual approach, and reiterating the benefits of knowing one’s HIV status. In addition, dental professionals felt that a discrete opt-in question offering HIV testing could be adapted into the standard medical history form, thereby avoiding uncomfortable conversations with patients. Most dental patients confidently reported that they would not personally be offended by the offer of an HIV test in a dental practice and explained that people might be grateful for the opportunity to test. There was agreement among most participants, excluding PWH, that oral fluid POCT readily fitted with the context of a dental practice. PWH worried that oral fluid tests might lead to misconceptions about modes of HIV transmission. Oral fluid tests were perceived as highly acceptable, avoided the need for finger prick, aligned with the dental professionals’ role within the mouth, and easily administered during a dental visit. However, the most important feature of the test for all participants was diagnostic accuracy. 

### 3.6. Establishing New Processes to Deliver HIV Poct within Existing Systems

Fundamental training needs for dental professionals included communication skills in several different domains: explaining the purpose of the study, approaching patients with an HIV test offer, managing a reactive result/breaking bad news, and managing a false-positive result. In addition, some dental professionals wanted a script to support the testing process. PWH suggested an update on current concepts in HIV, including U = U. Some dental professionals wanted to role-play clinical scenarios of managing reactive HIV test results. A frequently asked questions document was considered a valuable component of training. Additionally, protocols that included guidance on information sharing processes, signposting, instructions for documenting testing in clinical notes, and referral pathways were requested. 

A summary of the suggestions to practically achieve the integration of HIV testing into the dental clinic is presented in Table 3. Dental professionals commonly suggested dedicated walk-in sessions for HIV testing, similar to flu vaccination clinics in general medical practice. However, separate clinics were anticipated to lose the intervention’s opportunistic benefit, and patients were thought to be unlikely to attend these clinics. Dental professionals felt that for the opportunistic testing to be effective, you had to “strike while the iron is hot” and integrate it into the workflow of the appointment. Most dental professional participants concluded that offering and performing HIV testing within the same appointment was likely to yield greater uptake than a separate clinic or a deferred approach. However, they did acknowledge that this approach may be more disruptive to clinic functioning. Providing HIV testing information on a different day to performing HIV testing or providing information by post or electronically before appointments created a cooling-off period that could reduce uptake. 

Overall, dental professionals felt that the most appropriate strategy involved informing patients about HIV testing at reception upon arrival at the practice and having a tick box present on the medical history form. The test would then be offered verbally in surgery to those identified by the medical history response and performed at the end of the clinical appointment once dental treatment was complete. Dental patients felt that it would be most appropriate to limit the testing to assessment and hygiene appointments because it minimised the burden of having both dental treatment and testing in the same appointment. Additionally, offering testing to existing rather than new patients helped dental professionals anticipate how patients were likely to respond to the test offer.

## 4. Discussion 

The focus groups explored the acceptability of HIV testing from the perspective of UK-based dental professionals, dental patients, and PWH. Overall, dental professionals, dental patients, and PWH who took part in the focus groups felt that HIV testing in the dental setting was acceptable and of value to populations at high risk of HIV. However, questions arose about the appropriateness of the intervention in the dental setting context.

Most dental professionals who took part in the focus groups demonstrated a preference for oral fluid testing processes. However, test accuracy was considered the most important feature for almost all dental professional and dental patient participants. Blood-based rapid diagnostic tests have consistently shown higher sensitivity and specificity than oral fluid tests [29,30,31,32]. All focus group participants felt that it was important that HIV testing did not impinge on the time available for dental care. Studies in UK general medical practice have opted for the INSTI^®^ rapid finger-prick HIV test kit because of the rapidity of obtaining a highly accurate result in a time-constrained clinical setting [2]. 

Ensuring patients did not feel obligated or coerced to test for HIV was a key theme arising from the focus groups. In the present study, adopting a positive attitude around HIV testing and using patient-initiated opt-in approaches were suggested as ways to uphold patient autonomy in the decision-making process. Nonetheless, opt-out provider-initiated HIV testing is the recommended approach to delivering HIV testing in the UK and can encourage earlier HIV testing and diagnosis [33,34]. Further, in the UK, opt-out testing expansion plans are imminent, with more than GBP 20 million pounds ringfenced to eliminate the transmission of HIV by 2030. The National Institute for Health and Care Research plan to increase opt-out testing for HIV and Hepatitis B and C in 48 sites in areas of high HIV prevalence [35]. 

Across all focus groups, a universal approach to HIV testing was considered appropriate. In the UK, a universal (as opposed to targeted) approach to testing is recommended in areas where the population prevalence of HIV is ≥0.2% among 15–59-year-olds [10]. Adopting universal screening leads to more test offers and, thus, a higher testing rate than targeted testing. A further benefit of universal testing is that it circumvents HIV stereotyping [36,37] 

Limited physical space available in dental settings was identified as a challenge to ensuring discretion and confidentiality for HIV testing. PWH in this study and others report issues of lack of privacy in dental reception areas and fear of inadvertent disclosure of HIV status [20]. Focus group participants strongly felt that an HIV test offer integrated into the medical history form would enhance privacy. HIV testing guidance recommends face-to-face delivery of results in a confidential space [33]. 

Dental professionals in this study were worried about offending patients and uncertain about their competency to provide HIV testing and manage reactive HIV test results. The focus groups generated novel solutions to mitigate the risk of patients being offended or surprised when encountering HIV testing at the dentist. These included a range of tactics used to prepare patients for the offer of HIV testing, e.g., emails, text messages or a cooling-off period between appointments. However, these strategies are at odds with the intervention’s intended opportunistic approach. 

Dental professional focus group participants identified that their HIV knowledge was outdated. Despite global campaigns to promote U = U, healthcare professionals in general still have anxieties about committing to the U = U message, preferring to describe the risk as “close to zero”, “minimal”, or “low risk” [38]. Therefore, an update in general concepts around HIV was considered an essential component of HIV testing interventions. 

All dental professionals in the study were confident about providing dental care for PWH, and none anticipated that PWH should be referred for dental care at specialised services. Indeed, many participants commented that dental care should be provided for PWH as for any other patient, using the same universal cross infection control procedures. These findings highlight changes to dental professionals’ perspectives of the perceived “dangerousness” of HIV when compared with earlier studies from the 1990s. In these studies, most dentists wished to “double glove” and wear gowns to provide dental care for PWH and for PWH to be treated in specialised hospital services [19]. However, there was still an awareness that HIV stigma existed, posed a present concern, and could affect the willingness of the patient population invited to participate in HIV testing in dental settings. HIV stigma, as opposed to the transmissibility of HIV, caused the greatest anxiety for HIV testing in dental settings in this study. 

Some participants were concerned that vulnerable patients might fail to link to HIV care after receiving a reactive test result. Rapid point of care tests, professional training, and inter-professional collaboration are known strategies that improve linkage to HIV care [39,40,41]. Therefore, any HIV POCT interventions should feature well thought out referral protocols. 

The findings of this study are consistent with literature from the United States and Canada. A mixed-methods systematic review by Doughty et al. described key dental professional barriers to HIV testing as including narrow perceptions of the scope of practice, operational challenges, business tensions, and beliefs that HIV testing could negatively impact the patient–practitioner relationship [42]. Similar to the systematic review, this study highlighted that dental professionals in the UK would also be reluctant to discuss HIV and test results with patients. This is a key issue underpinning the success of any HIV testing intervention: dental professionals must be willing to discuss HIV and provide results of point of care testing in order for any HIV testing intervention in dental settings to be effective. Both in the UK and elsewhere, training for dental professionals on how to negotiate uncomfortable conversations is essential. Nevertheless, apprehension towards discussing HIV and a lack of HIV knowledge is not unique to dental practice; it is also a key barrier to general medical practitioners offering HIV testing [43]. 

In the US and Canada, facilitators of HIV testing included maximising the fit of the intervention within the dental setting context [42]. Within our study, the dental professionals clearly highlighted ways in which HIV testing could be integrated into UK dental settings. Akin to studies from the US and Canada, dental patients were overwhelmingly positive about the intervention provided it was performed by adequately trained staff and appropriate systems were in place for managing reactive test results. 

Senior dental nurses showed a willingness to provide HIV POCT and highlighted relevant additional skills that supported their involvement. However, motivation to engage did not extend universally to all dental nurse focus group participants; less experienced dental nurses were reluctant to deliver testing as they were uncertain about their competency. Despite patient acceptance of the enhanced duties of dental nurses and dental hygienists/hygiene therapists in the UK, skill-mix has not been fully realised in general dental practice [44]. This may have onward implications for the feasibility of HIV testing in UK dental settings and the cost-effectiveness of the intervention when compared to other healthcare settings. The qualitative data analysed in this study were subsequently combined with the findings of a systematic review of HIV testing in dental settings to form the evidence base for an HIV testing intervention. 

### 4.1. Strengths 

The findings from the focus groups advanced the literature in four ways. Firstly, the study was unique in exploring the views of participants recruited in the UK. Secondly, the study is the first to report the views of PWH about their attitude towards HIV testing in dental settings. Thirdly, this study takes forward the literature by including the views of patients who attended private dental practice. In the existing literature, qualitative research has only been undertaken with patients attending dental hospital settings. By including patients from diverse settings, this study provided insight into the impact of different dental contexts on the perceived acceptability of HIV testing. The final novel contribution included insights into the perspectives of dental nurses and their willingness to engage in delivering testing. Published qualitative literature has only included dentists, dental hygienists, and dental or hygiene students as interviewed members of the dental team. The study finds strength in the diversity of voices within the sample, including those that are seldom heard in dental research, such as dental nurses. 

### 4.2. Limitations

The focus group samples were not as diverse as intended. There were more female dental professionals included in the sample than originally proposed, with greater numbers of dental nurses and fewer dental hygienists. Trainee dental nurses were uncomfortable contributing to the discussion; contrastingly, more senior dental nurses actively contributed. Therefore, the views of trainee dental nurses are underrepresented in the study. Most dental patients and PWH were between the ages of 25 and 59 years; few participants were under the age of 25 or aged 60 years and over. Additionally, the voices of ethnic minority groups may not have been captured as the sampling strategy did not selectively recruit participants based on their ethnicity. Other limitations included dental patients and professional participants being selected from dental clinics that had agreed to participate in a subsequent HIV testing interventional study, which may have had an impact on the perspectives of the participants. Participants who agreed to participate in the study may have different perspectives from those who declined to participate in the study. Though the findings from this study may have transferability to other dental settings within England, the findings may not be reflective of the views of dental patients and professionals internationally. 

## 5. Conclusions

Dental professionals, patients, and PWH had mixed opinions about the feasibility and acceptability of HIV testing in the dental setting. Key barriers to HIV testing included logistical challenges, patients feeling surprised at being offered an HIV test during a visit to the dentist, and dental professionals’ anxiety about managing the results of reactive HIV tests. Communication skills for dentists were a key training need for the implementation of any HIV testing intervention. Clear operating procedures and protocols were essential. Strategies proposed to support the integration of HIV testing into dental settings included adequately preparing patients, team working, minimising the burden of the intervention, maintaining the focus of appointments on dentistry, maintaining discretion, and preserving the patient–practitioner relationship. Contemporary HIV testing strategies in the UK, specifically opportunistic and opt-out approaches, were at odds with the approaches to informed consent in UK dental practice culture. Careful consideration must be given to developing strategies to seamlessly integrate HIV testing intervention into dental settings with minimal disruption to usual workflow in order for such interventions to be successful. 

## Figures and Tables

**Table 1:** Descriptors of the dental patient and PWH participants.

Participant Descriptors		Participants Recruited
Gender	Male	12
	Female	7
Age group	18–24	2
	25–59	11
	60+	6
Participant	PWH	7
	Dental patients without HIV infection	12
Dental setting	Community Dental Service	3
	General Dental Practice	3
	Private dental practice	4
	Hospital	2
	Not applicable	7

**Table 2:** Dental professional participant demographic details.

Participant Descriptors		Participants Recruited
Gender	Male	8
	Female	32
Years since qualification	0–10	17
	More than 10	19
	Not disclosed	1
	Not applicable	3
Professional role	Dental nurse	15
	Dental hygienist	1
	Dentist	21
	Reception/administration	2
	Other	1
Dental setting	CDS	10
	NHS GDP	9
	Private GDP	13
	Hospital	8

**Table 3:** Recommendations for HIV testing in UK dental settings.

Suggestion	Details
Adequately prepare patients	- Send patients emails, text messages or letters with information. - Offer HIV testing at a separate appointment to perform testing.
Team working	- Work collaboratively with dental nurses and receptionists. - Identify a dedicated member of the dental team who was responsible for undertaking all of the HIV testing. - Have additional research staff on-site to deliver the HIV testing.
Minimise the clinical burden of the intervention	- Build HIV testing into the regular examination or hygiene appointment. - Host a dedicated separate clinic for HIV testing out of hours or available on the weekend. - Performing the test within a dental appointment but deferring the management of test results to a sexual health team. - Offer POCT only during appointments before lunch or at the end of the day. - Provide HIV self-tests in the dental setting. - Limit the testing to assessment and hygiene appointments. - Personalised frequency of test offers could be adapted for each patient based on risk.
Maintain the focus of the appointment on dentistry	- Offer POCT at the start of the appointment and perform it at the end of the appointment after treatment is complete. - Use “dead space” while radiographs are being developed.
Maintain discretion	- Offer HIV testing on the medical history form, thereby avoiding conversations about HIV unless a patient has responded affirmatively.
Preserve the patient–practitioner relationship	- Offer testing to existing rather than new patients.

## Data Availability

Anonymised transcripts from the data can be obtained from by emailing the lead author Janine Doughty (janineyd@liverpool.ac.uk).

## Supplementary Materials

The following are available online at https://www.mdpi.com/article/10.3390/dj12080246/s1, Table S1: Coding strata.
