# Supplementary material for: Exploring the Acceptability of HIV Testing in the UK Dental Setting: A Qualitative Study
Source: Dent J (Basel). 2024 Aug 2;12(8):246. doi: 10.3390/dj12080246 (PMC11352954; doi:10.3390/dj12080246)
Supplement: Supplementary file 1 [file dentistry-12-00246-s001.zip › dentistry-2972755-supplementary.pdf]

Supplemental Table S1: Coding strata

Level 1	Level 2	Level 3
Theme 1: Perceptions of HIV and implications for POCT acceptability	Current concepts in HIV	Knowledge about HIV
		Universal standard of care
		Attitudinal changes
		Destigmatisation
	HIV stigma	HIV stereotypes
		Perceptions of HIV risk-taking behaviours
	Cultural shifts in perceptions of sexuality	Gender and sexuality norms
	Public health priorities	Improved health outcomes
		Other health priorities
		HIV absent from media
		Sources of information about HIV
Theme 2: Understanding the purpose of HIV POCT in the dental setting	Opportunistic benefit	Patterns of dental attendance
		Convenience
		Discrete approach to HIV testing
		Absence of symptoms
	The perceived value of knowing ones' HIV status	Timely access to treatment
		Reassurance
		Dental professional safety

**Level 1**	**Level 2**	**Level 3**
	Reframing HIV testing	Dental role<br>Holistic practice
Theme 3: Appropriateness of dental professionals to provide HIV POCT	Patient-practitioner relationship	Trust and familiarity<br>Confidentiality<br>Duty of care<br>Perceptions of the dental professional<br>Disclosing HIV at the dentist
Theme 4: Appropriateness of the dental environment for HIV POCT	The fit of HIV testing with the purpose of the dental setting	Dental practice culture<br>Existing skills
	Competing priorities for dental practice resources	Dental treatment as the priority<br>Keeping to time
	Dental practice environment	Team working<br>Inter-professional working<br>Physical space<br>Confidentiality
Theme 5: Acceptability of the POCT intervention in dental settings	Approaches to offering an HIV test	Approaches to test offer<br>Discretion and privacy<br>Team member responsible for HIV testing<br>Informed consent

Level 1	Level 2	Level 3
		Anticipated patient response
		Feelings about test offer
	HIV test unit	Testing medium
		Accuracy
		Process and timing
	Managing HIV test results	Referral processes
		Breaking bad news
		Vulnerable patients
		Professional distress
Theme 6: Establishing new processes to deliver HIV POCT within existing systems	Training and education	NA
	Preparing patients and normalising HIV testing in the dental setting	NA
	Seamless integration	NA
